# GTSO: Global Trace Synchronization and Ordering Mechanism for Wireless Sensor Network Monitoring Platforms

**DOI:** 10.3390/s18010028

**Published:** 2017-12-23

**Authors:** Marlon Navia, José Carlos Campelo, Alberto Bonastre, Rafael Ors

**Affiliations:** 1 ITACA, Universitat Politècnica de València, Camino de Vera, s/n, 46022 Valencia, España; jcampelo@itaca.upv.es (J.C.C.); bonastre@itaca.upv.es (A.B.); rors@disca.upv.es (R.O.); 2 Carrera de Computación, Escuela Superior Politécnica Agropecuaria de Manabí (ESPAM MFL), 10 de Agosto and Granda Centeno Street, No. 82, 130601 Calceta, Manabí, Ecuador

**Keywords:** traces synchronization, offline synchronization, monitoring platforms

## Abstract

Monitoring is one of the best ways to evaluate the behavior of computer systems. When the monitored system is a distributed system—such as a wireless sensor network (WSN)—the monitoring operation must also be distributed, providing a distributed trace for further analysis. The temporal sequence of occurrence of the events registered by the distributed monitoring platform (DMP) must be correctly established to provide cause-effect relationships between them, so the logs obtained in different monitor nodes must be synchronized. Many of synchronization mechanisms applied to DMPs consist in adjusting the internal clocks of the nodes to the same value as a reference time. However, these mechanisms can create an incoherent event sequence. This article presents a new method to achieve global synchronization of the traces obtained in a DMP. It is based on periodic synchronization signals that are received by the monitor nodes and logged along with the recorded events. This mechanism processes all traces and generates a global post-synchronized trace by scaling all times registered proportionally according with the synchronization signals. It is intended to be a simple but efficient offline mechanism. Its application in a WSN-DMP demonstrates that it guarantees a correct ordering of the events, avoiding the aforementioned issues.

## 1. Introduction

During the last decades, the evolution of computer systems is heading in the general direction of distribution of processing elements. From primitive mainframes to the personal computers (PCs) in the 80 s, this century has started with the explosion of mobile telephony and next steps leading to a new environment, the Internet of Things (IoT), where computers disappear inside current objects to create intelligent objects. This evolution has been possible thanks to two main advances: the evolution of processor systems, cheaper and smaller, but more powerful as time goes by, and the incredible advances in communication networks, which provide better communication services and worldwide coverage.

Context awareness, the ability to detect changes in the environment and react in consequence, is one of the main issues in IoT systems. An ecosystem of intelligent objects that collaborate autonomously to satisfy the user requires being receptive to environmental modifications, adapting and even predicting these changes. The best tools to provide context awareness are the so-called Wireless Sensor Networks (WSN). Many ad-hoc WSN developments have been proposed, but new advances in this field point to open-standard based WSN deployments [1]. As a Distributed System (DS), WSNs are difficult to evaluate, monitor and debug. These analyses may be performed by non-standard ad-hoc methods when the operation of WSN is not critical (i.e., leisure applications, ambient comfort, etc.). Nevertheless, when the WSN applications involve health, vital supplies or industrial production, restrictions such as fault tolerance, security, safety and robustness must be granted. Thus, in these environments, reliable standard analysis techniques must be provided [2].

In this line, the most useful tools for WSN monitoring—and even for monitoring any DS—consist in the so-called *monitoring platforms*. Many of these platforms—which may be based on hardware or hybrid approaches—include a set of dedicated nodes, called *monitor nodes*, which have to be added to each one of the WSN nodes to monitor. A monitor node must obtain information about the performance and state of its attached WSN node. Other approaches also use a set of *sniffer nodes* that are in charge of listening and recording information about messages transmitted by the nodes. All the information regarding WSN nodes must be processed in a centralized manner in order to foresee a view of the whole system.

Many DMP for WSN—WSN-DMP—have been proposed [3,4,5,6,7,8,9,10,11,12,13]. These proposals may be classified according to several criteria. It is very common to divide them into *online* and *offline* approaches. The first ones reduce the latency during diagnosis and can provide a deeper view of the WSN behavior, but increase the overload. Offline monitoring approaches are based on the generation and recollection of logs (traces), which permit later analysis, or reconstruction of the WSN performance. In this line, debugging by reproducing the behavior, through the use of traces, is a powerful tool to detect failures [14]. 

Offline monitoring proposals may be based on hardware or software approaches [15]. Most of them, however, consist of distributed platforms where monitor nodes acquire time-related information in an individual manner—as traces or logs—to analyze data. Time is included by means of a timestamp, which records the value of the local timing when the event has occurred. For processing, traces have to be centralized into a single device—which acts as a server—to be processed. Trace processing is a topic that has been widely studied in centralized monitoring systems. However, logs or traces generated by monitoring platforms nodes must use a common reference time before being interpreted. Otherwise, it is not possible to establish relationships between the events registered in different nodes. 

The synchronization mechanisms can be focused on synchronizing clocks of the monitor nodes, or may centered directly on the traces. The time synchronization between nodes in a DS is a classical problem that has been widely studied. Internal clocks can suffer deviations due to hardware issues or environmental conditions such as temperature [16]. Mechanisms focused on nodes’ clocks synchronization may be applicable, however, this solution is not always the best. A “back in time” problem may arise when synchronizing traces: when the local clock of a node has been running faster than other clocks in the platform, and they are all corrected to a common value, later events may be included in the log with inconsistent timestamp values. These errors may also change the order of some events logged locally, thus hiding cause-effect relationships, and leading to false conclusions after analysis [17]. 

Trace synchronization can be online or offline. The difference between these two synchronization approaches is similar to that explained for monitoring. The online traces synchronization mechanism works in streaming mode, generating the adjusted or corrected timestamp while the trace is generated. In this way, a synchronized trace is obtained directly from the monitoring platform. 

Offline synchronization performs the trace synchronization after the end of tracing generation and all traces have been collected. The offline approach applies mathematical models algorithms for estimating clocks offset and drift [18]. In this approach, the traces may be synchronized applying slower but more precise mechanisms. 

The authors of this paper have previously proposed an offline distributed monitoring platform for WSNs using a hybrid approach. This platform needs to apply a synchronization mechanism to the obtained traces, in order to provide coherence for the analysis. To overcome undesired behaviors without having to perform many calculations or impose any restriction on the system to be monitored, this paper proposes an alternative and simple but efficient trace synchronization method, based on the generation of common synchronization points, called GTSO: Global Trace Synchronization and Ordering mechanism. This mechanism performs offline timestamps adjustments or corrections by using global synchronization events.

The rest of the paper is organized in the following way: in Section 2 the hybrid monitoring platform is described, and a more detailed description of the issues that current synchronization methods may cause in traces is made. Later, Section 3 summarizes the state-of-art in both WSN-DMP operation and synchronization, as well as in most relevant proposals in synchronizing traces. Section 4 presents a new proposal for global trace offline synchronization in a DMP. Section 5 presents the tests that have been proposed to evaluate the mechanism and its results. Finally, Section 6 presents the obtained conclusions.

## 2. The Trace Synchronism Problem in DMP

Trace-based monitoring systems are abundant in the literature, especially in centralized monitoring systems. Monitors record the sequence of events that are considered relevant in the system to be observed. Each event is stored with a Timestamp information that reflects both the sequence and elapsed time between events.

As indicated in the introduction, when monitoring a DS as a WSN, it is necessary to perform parallel trace captures in each one of the Monitoring Platform components. In [2] is proposed an architecture for the systematic study of these platforms, and described in detail the most relevant ones to date. As a common aspect of most of these platforms, a set of monitors acquire information from the system in a distributed way, and then analyze it. 

**Figure 1:**
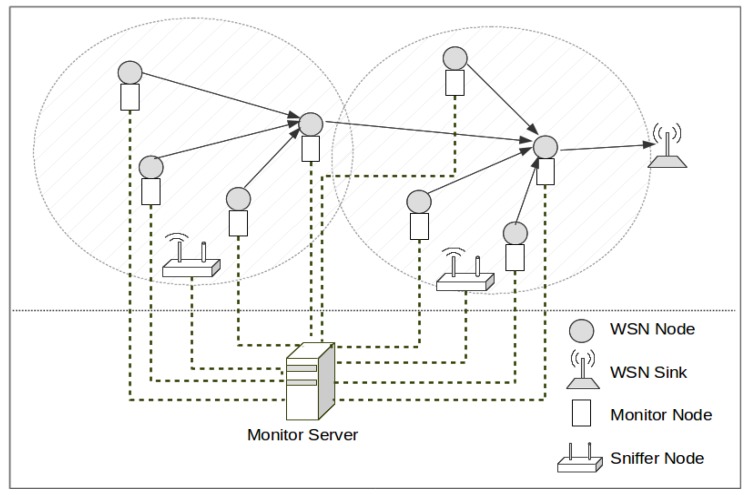
Diagram of a hybrid monitoring platform proposed by the authors.

In the WSN-DMP proposed by the authors in [13], this treatment is done centrally, merging the obtained traces to be able to reflect the overall behavior of the system. This platform is composed of monitor nodes, and sniffer nodes. They may use a separate network—apart from the monitored network—for collecting traces, as is shown in Figure 1, or store the logs in non-volatile memory and manually collected for later processing. Monitor nodes are attached to WSN nodes, and register events directly from them with very little intrusion. Sniffer nodes capture WSN transmissions on the air. All traces generated by monitor and sniffer nodes are centralized in a Monitor Server, for merging and analysis.

This is a hybrid monitoring platform that combines both active approach (monitor nodes) and passive approach (sniffer nodes). Obviously, this process requires synchronizing the timestamps of their traces. Without a correct synchronization, a correct fusion of traces is not possible for later processing—because the order of events can be altered—and therefore the behavior of the system cannot be properly studied.

Usually, many synchronization mechanisms for DS force the clocks of the nodes to adopt a common time at the same moment, called synchronization point, or estimate drift to correct the node clock time. After a synchronization point, all nodes are supposed to be synchronized, sharing a common time base. Some of the WSN-DMPs, that are mentioned in the next section—specially those that use NTP—are included in this category. The application of this kind of synchronization mechanism can result in errors when timestamps are generated, because the rate of the clocks in the nodes can be faster or slower than the reference clock. When these mechanisms are applied to the monitoring platforms, the Timestamp of the captured event is affected. The intervals between events can change, and even the order of their registration may be altered. Synchronization mechanisms for DS do not ensure avoiding event order changes, because they are focused on nodes synchronization [19]. WSN synchronization has been widely studied, and there are proposals that offer high accuracy and precision for drift estimation and correction [20]. However, these proposals still can induce event order inversion.

**Figure 2:**
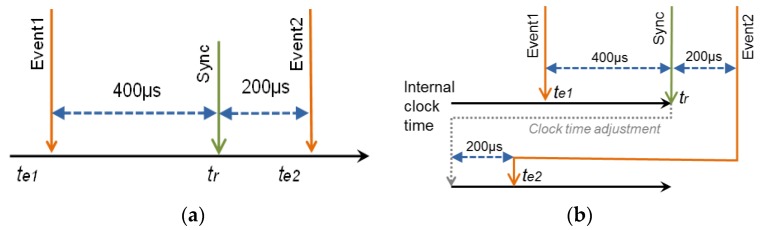
Almost simultaneous events and synchronization message, (**a**) with perfect clock (no) adjustment; (**b**) With erroneous internal clock (Set adjustment).

As an example of this effect, consider the case of a platform where a monitor node registers two events (*event1* and *event2*) that happen sequentially with a time between them of 600 µs (Figure 2). In the case of a clock without offset, the synchronization point does not change the value of the node clock. In this example (Figure 2a), a synchronization point arrives 400 µs after *event1*, and 200 µs before *event2*. Despite the closeness of these events, the timing of the recording is correct. 

Let us suppose that, in the same sequence of events, the internal clock of the monitor node accumulates an offset of +700 µs from the last point of synchronization. Figure 2b illustrates what happens in this case. *Event1* occurs 400 µs before the synchronization point, and it is recorded with a time (*t_e1_*). After 400 µs, the synchronization point *t_r_* arrives. The monitor node realizes that its time should be 700 µs earlier, and decrements its internal clock 700 µs. When *event2* happens, 200 µs later, Timestamp of *event2* (*t_e2_*) should be *t_e1_* + 600 µs (or *t_r_* + 200 µs). However, as the internal clock was changed backward 700 µs, *t_e2_* would appear to be earlier than *t_e1_* (in fact, equal to *t_e1_*–100 µs). When traces are processed, *event1* seems to happen after *event2*.

In the case of a real monitoring platform, the effect is multiplied by the existence of numerous monitor nodes, as many as nodes in the WSN, plus the sniffer nodes. In this context, it is common that many related events may be observed in two or more elements of the WSN-DMP. The timing errors that have been described in a single trace processing become more frequent when dealing with different timestamp timing. Clocks rate difference and drift has already been studied and evaluated in [21]. Thus, time lapses between events in different nodes, and even its sequence, may be inconsistent because of this phenomenon. These inconsistencies in the traces can make that the reproduction of the behavior of the WSN, mainly for debugging purposes, is inaccurate.

**Figure 3:**
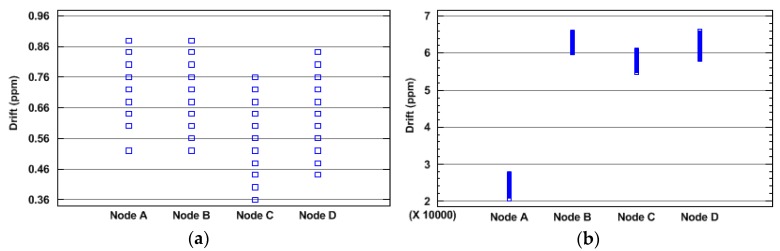
Scatter diagram of drift variation in four nodes (**a**) with a high quality clock source, and (**b**) with a low quality clock source.

On the other hand, in [21] another issue was detected: the clock rate is not always stable, even when a high quality clock source is used. The influence of environmental factors—for example the temperature—over clocks skew have been analyzed [16,22]. Figure 3 shows the scatter diagram of measured drifts of the clocks of four nodes with the same hardware for both low quality and high quality timing sources. The drift was calculated in time intervals of 10 s, during one hour of test. As can be seen, the drift can vary up to 0.4 ppm (parts per million) from lowest to highest value for the same clock with a high quality clock source (Figure 3a). Due the reduced values obtained, there are repeated values represented in the Figure 3a. This may cause clock variations of tens of microseconds in 60 s. In the case of low quality clock source, the drift variation reaches almost 10,000 ppm of variation (Figure 3b). For long time periods, it can result in a considerable drift difference between two different time intervals. In this case, variations may reach 600 ms each 60 s.

**Figure 4:**
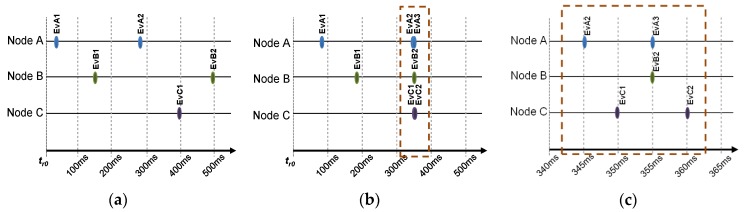
(**a**) Sparse event generation; (**b**) Almost simultaneous events; (**c**) Closer look to interval 300 ms–400 ms in (**b**), with higher precision.

Granularity is another main aspect to be considered about the trace generation. If many events are registered with the same Timestamp, they will be considered simultaneous. When the occurrence of events is sparse (Figure 4a), their sequence can be correctly registered. But when many events occur in a short period of time (Figure 4b), its Timestamps may not define the correct sequence of time, and they look as if they happen at the same instant of time. This may be irrelevant in some applications, but not in others. To reflect the correct sequence of Figure 4b, the granularity of the Timestamps must be refined to offer a smaller period, as long as the timestamps clock allows it. Figure 4c shows how the increment of granularity—reducing the timestamp clock period—would provide better time resolution and thus these events are correctly registered. Obviously, this solution is limited by the characteristics of the hardware clock used for monitoring. 

When the generation of events is sparse enough—as shown in Figure 4a—a small drift of a few milliseconds is tolerable without affecting the sequence of events in the traces. However, Figure 4b shows that many events occur in a short period. In this case, a small difference in synchronism of clocks may change the order of the events in the trace. Figure 4c shows a closer look to the set of simultaneous events in Figure 4b. As stated above, the exact sequence of events may be very important, as an event sequence may be cause of the malfunction of a WSN. Additionally, timing issues between events, such as retransmission time, may also be relevant for WSN analysis and debugging. 

The best option to overcome all the aforementioned issues would be the use of a common clock source—e.g., a crystal oscillator—in all the nodes of the monitoring platform. Unfortunately, this option is usually not possible when monitoring deployed WSN in real environments. Thus, this solution is limited to those platforms applied to the monitoring of laboratory experiments. Therefore, a more realistic option is to allow each component of the monitoring platform to use its own local clock for Timestamp of events, assuming that traces must be synchronized in later processing. 

In brief, according to the previous analysis, a traces synchronizing method for a WSN-DMP should:

- Manage different drifts and offsets of monitor nodes and all components of the monitoring platform.
- Achieve the higher possible precision for registered Timestamps.
- Sort the events registered in traces correctly.
- Be applicable to every element of the monitoring platform.
- Cause the lowest possible interference, so it must be as simple as possible.
- Be easy to implement, independent from the communication protocol and not requiring the modification of the observed system.

Thus, our proposal consists in a mechanism to synchronize traces without changing the local time of the monitor nodes. This mechanism actuates asynchronously, in a post-process of the traces, as far as common events that permit establishing a relation between Timestamps in traces are available. This proposal is oriented to offline synchronization—although the main idea could be applied to online implementations—and needs a broadcast network with low and/or constant delay.

## 3. Related Work

This section details the state-of-art about synchronization procedures in WSN-DMP. A summarized description of these WSN-DMPs is followed by a study of the synchronization procedures for these platforms. 

### 3.1. Distributed Monitoring Platforms for WSN

Let us suppose that there is a WSN, composed by sensor and sink nodes, to be observed. The main idea of a WSN-DMP consists on deploying an additional set of monitor nodes (that may also be connected by means of a secondary communication network). Each monitor node can be attached to a sensor node—and may obtain data from its inner operation—or simply be designed to capture all transmissions (sniffer nodes) from the observed WSN. The captured information usually is centralized and processed in a server. The main differences between WSN-DMPs are related to what information is captured and how it is processed. 

SNIF [3] deploys a network of nodes, called *sniffers*, which capture the WSN transmissions in the air. These monitor nodes transmit—via a Bluetooth interface—the captured data tagged with a timestamp to other device in order to process it. This device works as a sink: receives the packets and processes information. Its authors have been developed a framework for analysis and debugging. 

Pimoto [6] is similar to SNIF. It also deploys a network of nodes—which are called monitors—and captures the WSN transmissions in the air. They also use Bluetooth to deliver the captured data to a *Gateway*, which is a computer that tags the packets with a timestamp and forwards them, via TCP/IP, to a central server for analysis. Pimoto can also work over more than one sensor network simultaneously. Its authors have created a plugin for Wireshark—a traffic analysis tool—in order to visualize and analyze captured data. 

DSN [4] is a kit for testing and monitoring WSN applications. It is composed by a set of nodes attached to sensor nodes, all of them connected via Bluetooth to reach a server. DSN nodes record events transmitted by node application—through one of its serial interfaces—and send the information to the DSN server. Additionally, they can also serve as infrastructure for remote programming, transmission of commands, and dynamic configuration of monitoring.

LiveNet [5] merges the traces obtained by sniffers—connected to a host, as a PC—in order to provide information about dynamic operation of sensor network. The analysis is performed offline, after capturing all WSN transmissions.

SNDS [7] works in a similar way that SNIF or Pimoto, but it uses an Ethernet connection instead of Bluetooth. TCP and UDP protocols are used for data transmission and synchronization respectively. SNDS is focused in real time data capturing and analysis. 

In NSSN [8], sniffer nodes can detect automatically the work frequency of the target WSN to observe. The collected data is sent via wireless links to a monitor server, which parses, pre-processes, and stores data in a database. The information stored in the server can be remotely accessed by clients, which can observe or analyze it. 

EPMOSt [11] is another WSN-DMP focused on the reduction of energy consumption of monitoring network. There are local monitors—as in Pimoto—that collect information from sniffers and transfer it to a server. This system provides information using a SNMP (Simple Network Management Protocol) agent.

Testbeds for WSN allow debugging and monitoring in a controlled environment. Minerva [9] is not a monitor, but a testbed for WSN. It uses a debugging and tracing port connected to the sensor node to observe the behavior of the node. Minerva monitor nodes are connected through an Ethernet network. A server controls the platform and collects data from nodes. FlockLab [10] is also a testbed, but its platform components can use Ethernet or wireless connection. FlockLab can use others interfaces different of used by Minerva. In [12], an industrial oriented testbed—TWECIS—is presented. It combines features as tracing and actuation, debugging possibility, accurate synchronized power measurements; in terms of timing synchronization.

Therefore, most of WSN-DMPs merge the data obtained by the monitor nodes in a server. This server can perform analysis, visualization, or behavior reconstruction. So, the merged information should be synchronized with a reference time, for an adequate and precise analysis. WSN-DMP proposals describe in detail how to obtain data from monitoring. However—as explained bellow—they use mechanisms with low accuracy, potential inaccuracies risks, or that need expensive hardware, for synchronization.

### 3.2. Synchronization in WSN Monitoring Platforms

Not all the distributed monitoring platforms for WSN describe the synchronization mechanisms that they apply. They may use a standard or known protocol to do it, or they propose another way to carry out it, as explained below.

For example, the sniffer nodes of SNIF [3] use a synchronization mechanism developed by its authors in [23]. This mechanism exploits some characteristics and available functions of Bluetooth connection, by interchanging the offsets of Bluetooth clocks interfaces, and comparing them. However, it only reaches a precision of few milliseconds, and just works for Bluetooth nodes. 

LiveNet [5] tries to normalize—synchronize—the timestamps of several sniffers by taking one of them as reference timing. It divides the traces in intervals and calculates the offset of each interval, based on common and unique events—transmissions—registered in a pair of nodes. With the estimated offset, it corrects the timestamp for each interval. Since LiveNet needs these common events to perform synchronization, it is possible that traces of some sniffers cannot be synchronized adequately, because some sniffers may be unable to capture the same events than the reference sniffer, as its authors admit.

Many monitor nodes with TCP/IP capability—as Pimoto PC Gateway, sniffers of NSSN [8], components of Minerva [9] or FlockLab [10]—use the Network Time Protocol (NTP) for synchronizing. NTP performs synchronization by messages exchange with a NTP server, and it does not need more hardware than a network interface. However, it was designed for wide area Internet communications, so it reaches an accuracy of few milliseconds and may cause clocks of the nodes to go back in time [24].

Pimoto [6] uses a trick to synchronize traces from the monitor nodes: These count the milliseconds since they begin to work—using a counter of 4 bytes—and they put this parameter as a timestamp to the packages that they capture. The PC Gateway in Pimoto processes these “timestamps”, and converts them into a synchronized timestamp, based in the timing of the first package sent by the node. As these Gateways are synchronized using NTP, the accuracy and precision of traces synchronization are limited by this protocol. Besides, Pimoto’s mechanism does not consider that the clock rate can differ from one monitor to another.

Sniffers of SNDS [7] also dispose of TCP/IP stack, but they use Precision Time Synchronization Protocol (PTP) [25] to get synchronized. PTP is focused in measurement and control system, and provides a theoretical accuracy of microsecond to sub-microsecond. Nevertheless, PTP requires special hardware, and the synchronization update interval is around 2 s [26].

Components of TWECIS [12] exploit their backbone connection and use Real Time Ethernet (RTE) for synchronizing the measurements. The use of RTE is a very good alternative to get high accuracy synchronization, but involves additional or special hardware, which may be expensive.

Some platforms as DSN [4] mention the use of high accuracy timestamping protocols, but they do not describe how they work. Its authors only mention that the accuracy of this protocol is higher than the time-of-arrival of command broadcast messages, but not how they get it. Meanwhile, others platforms as EPMOSt [11] do not indicate anything about synchronization between its components, although they affirm to use timestamps in their operation.

Then, most of WSN-DMP performs an online synchronization between their nodes; and only a few—as LiveNet or Pimoto—performs a kind of offline synchronization of captured data. Therefore, inaccuracies on synchronization can occur due the explained synchronism problem. 

Besides, there are several algorithms for synchronization of traces generated by a DMP, both online and offline. The most mentioned algorithms are linear regression and convex-hulls [18]. Based on convex-hulls, others proposals for synchronization have been developed [27]. In addition, linear regression does not guarantee avoiding message inversions [28]. Although both were developed for offline synchronization, convex-hulls approach has been used in online synchronization [29]. Another offline mechanism uses kernel level events to perform synchronization [28]. Many of traces synchronization proposals—offline or online—are focused in high performance computation systems [30,31,32].

Despite the available algorithms and proposals of traces synchronization in DMP, we have not found a WSN-DMP—neither online or offline—that apply some of them, as explained. Therefore, it would be possible their implementation in this environment, specially for offline monitoring. On the other hand, we have proposed a simpler solution, which does not require special requirements, such high performance systems or large computers clusters.

## 4. Proposal for Global Trace Synchronization

Many conclusions arise from the previous analysis about WSN-DMP and traces synchronization protocols and mechanisms. In brief, the following aspects must be considered when designing an effective global trace synchronization protocol:

- Monitoring operation needs to synchronize the obtained information—traces—against a reference time. Without an appropriate trace order, the analysis can result in false conclusions.
- Mechanisms focused on node synchronization, which change the internal clock but do not correct the scale of clock offset or drift for correct them, must be discarded.
- In other environments, the synchronization of traces has been solved by using more complex methods, such those are based on linear regression or convex-hulls [18]. These mechanisms are required when synchronization has to be deduced from related events, such as transmission and reception of messages, which are separated by an unknown and variable amount of time. On the other hand, the traffic behavior of WSN (tree architecture towards a Gateway) does not cause enough related events to coordinate all the traces in the system. In addition, in the case of sniffers, with no transmission events, the application of these techniques is not straightforward.

Our proposal—GTSO—applies an offline synchronization procedure (*post-facto* synchronization). It is based on the inclusion of global common events (synchronization points) in the traces that monitor nodes (or sniffers nodes) register during the operation, along with the captured relevant events. These synchronization points do not interfere with the observed WSN, because they are transmitted through alternative mechanisms, such as the monitoring network, that broadcast the synchronization point in a way that is received simultaneously in all the monitor nodes. In each monitor, the arrival of synchronization points is registered in the local trace with its local Timestamp. This will make possible to correct the timestamps of the events registered in the traces. 

GTSO may be considered a hybrid approach between online synchronization of the monitor nodes and offline processing of traces. Therefore, there is no common clock for all the nodes in the DMP, but a periodic sync point is received, and registered, in all these nodes simultaneously. In this way, the performance of GTSO is only limited by the precision and variability of the time of register in the traces of this sync point, due to drift, drift variability and jitter.

There is a special node—called *SyncRoot*—which generates and sends the synchronization points periodically, by transmitting synchronization frames. Each synchronization point is numbered as unique, and the SyncRoot stores them in a log with the local timestamp. When a synchronization point arrives to monitor or sniffer nodes, it is registered in the local trace with its Timestamp, according with its local clock. When the monitoring experiment ends, all traces are delivered to the *Monitoring Server*, which could be also the SyncRoot server, and the trace synchronization procedure GTSO can be applied. This procedure corrects the Timestamps of each trace assuming that the Timestamp of each synchronization point in a node corresponds to the Timestamp of the same synchronization point in the Monitoring Server. The clock drift in that period is estimated, and the Timestamp of each event recorded between two synchronization points is recalculated proportionally according with that drift. If two synchronization points are sufficiently close, the clock rate variation in a node may be considered small enough to assume that the drift is constant. This enhances the relevance of synchronization period on the precision of GTSO: if the synchronization period is too large related to the clock stability, the drift variability may increase the error when synchronizing.

Finally, all the traces may be merged in a single sorted trace, which includes all events and their parameters, their corrected Timestamp, and the monitor node that provided each event.

**Figure 5:**
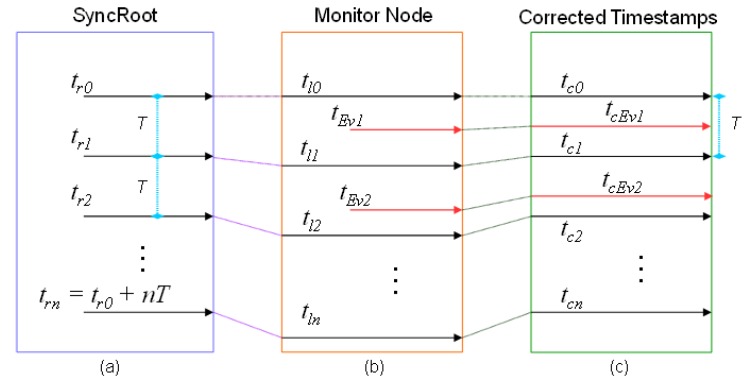
Schematic of global trace synchronization method. (**a**) The SyncRoot generates the sync points periodically; (**b**) The Monitor Node registers events and synchronization points; (**c**) The timestamps are corrected.

**Figure 6:**
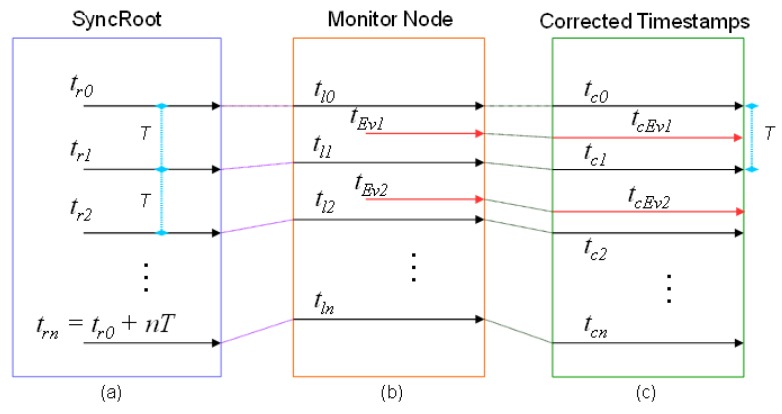
Schematic of global trace synchronization method. (**a**) The SyncRoot generates the sync points periodically; (**b**) The Monitor Node registers events and synchronization points; (**c**) The timestamps are corrected.

Figure 5 and Figure 6 show graphically the operation of the mechanism. In both Figures, the computer that assumes the role of SyncRoot transmits periodically a synchronization point (Figure 5a and Figure 6a). *T* represents the period of synchronization points. The time *t_r0_* is the reference time, at which the first synchronization message is sent, and *t_r0_ + nT* the time in which successive synchronization messages are sent. The monitor (or sniffer) node inserts in its trace a new event recording the arrival times of the synchronization point (*t_lx_* in the Figure 5b and Figure 6b), according with its local time. These Timestamps can be ahead or delayed compared with the reference time in SyncRoot. In the case of Figure 5b, the local clock rate is faster than SyncRoot (Timestamp values are greater than the SyncRoot time), whereas in Figure 6b the Timestamps are delayed, as the local clock rate is slower than SyncRoot. 

As previously stated, at the end of the monitoring operation, the traces are processed at the Monitoring Server. As seen in Figure 5c and Figure 6c, the times of the local synchronization marks *t_lx_* are matched with the *t_rx_* points of the SyncRoot, and are denoted as *t_Cx_*. After that, the *t_Evx_ Timestamps* of the recorded events are recalculated proportionally, according to the closer synchronization points (previous and posterior). New timestamps are denoted as *t_cEvx_* once recalculated.

The correction of Timestamps is performed by means of the following procedure: Let us *t_Evm_* denote the Timestamp of an event m, that has been recorded in a monitor node between two consecutive local synchronization points with timestamps *t_ln−1_* and *t_ln_* that’s it, *t_ln−1_ ≤ t_Evm_ ≤ t_ln_* with *t_ln−1_ < t_ln_*. The corrected Timestamp of the event *m*, denoted as *t_CEvm_*, may be calculated with Equation (1), where *t_Cn−1_* is the Timestamp in SyncRoot of the synchronization point recorded in *t_ln−1_*, and *T* is the transmission period of synchronization points:

(1) $$t_{CEvm}=t_{Cn-1}+\frac{t_{Evm}-t_{ln-1}}{t_{ln}-t_{ln-1}}T$$

As it was explained before, the SyncRoot numerates sequentially the generated synchronization points, and logs it with a defined format “<point>,<time>” (*####*, *hhmmss.μssec*) for later processing of traces. The first field of this format (*####*) refers to the sequence number of the point in hexadecimal notation, and is unique for each point in an experiment, whereas the time includes hours (hh), minutes (mm), seconds (ss), and microseconds (μsec). The sequence number of the synchronization point is sent to the nodes, which register the synchronization event—with its sequence number—in its traces, including its local Timestamp. In normal operation, two consecutive synchronization events in a monitor node correspond to two synchronization points in SyncRoot, so Equation (1) can be applied. However, if a monitor or a sniffer node loses one or more synchronization points, the trace synchronization still can be performed by applying the Equation (1) using the two synchronization points closer (before and after) to each event in the trace. In this case, the value of *T* in (1) must be the difference between these two synchronization points in SyncRoot. 

As the rate of drift is not stable over time, the value of the synchronization points transmission period (*T*) must be adjusted to the characteristics of the local clocks. As discussed in the next section, *T* can be increased or decreased according with the quality of the local clock source and the required accuracy.

The proposed solution assumes a very small propagation time of each of the synchronization points, the same for all the monitor nodes. It is also considered that media access and reception times are negligible. This is feasible because the secondary monitoring network has only one transmitter (the SyncRoot) for all synchronization point messages, and all the receptors use the same hardware. It is supposed to be a broadcast shared media, and the arrival of the message is the same in all the receivers (nodes). This network should not add additional delay due to network buffering or switching. Also, the synchronization events (points) at nodes must be handled with the highest priority. Besides, it does not need modifications of frames at MAC level, as others proposals do.

This secondary network may be used, after the end of the monitoring operation, to collect all the traces to the Monitoring Server.

GTSO even may be expanded to a tree structure. In a hierarchical DS, each monitor node may act as SyncRoot device for their child nodes. In this situation, a monitor node would receive and process the monitoring traces of the monitor nodes under it, and then transmit the joint trace to upper level nodes, until the traces arrive to the root of the tree. In this case it is required the addition of a level indicator to the synchronization points, as other proposals do.

## 5. Evaluation

This section describes the evaluation of the proposed mechanism GTSO. First, the hardware used for implementation and testing is described. Two different experiments have been considered. The first experiment evaluates the time precision of the mechanism, whereas the second experiment verifies the sequence of the recorded events. 

In these experiments, GTSO is compared with an internal clock adjustment approach that we call *traditional synchronization*. This mechanism is based on the replacement of the current value of the Timestamp clock when a synchronization frame arrives. No drift or offset are calculated or estimated for adjust timestamps. Both methods have been applied to the traces obtained in the experiments.

These experiments are listed and explained below.

### 5.1. Hardware Used and Configuration

**Figure 7:**
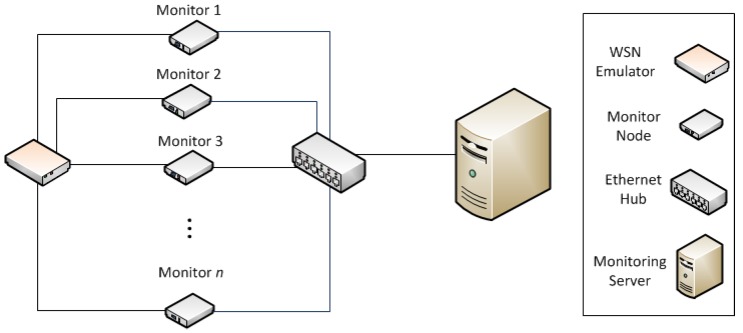
Architecture and elements of the experiment.

Figure 7 shows the hardware elements for the performed tests. Both experiments were performed using a monitoring platform that observes a simulated WSN. In this platform, the monitor nodes (from monitor 1 to monitor *n*) are based on a STM32F4 Discovery board [33], which includes a Real Time Clock (RTC), with an STM32F4DIS-BB expansion board [34]. The precision of the timestamps generated by the monitor nodes depends of the source of the RTC (oscillator or crystal, internal or external). 

In this board, like many prototyping environments, the RTC clock source can be internal (LSI) o external (HSE) to the microcontroller. An external crystal provides a better precision than the internal LSI. Using the external crystal, the highest frequency that can be applied to the RTC is equal to 1 MHz. With this frequency, the minimum quantum of the clock is equal to 40 µs [21], due the characteristics of the microcontroller. On the other hand, when using LSI (a 32 KHz RC oscillator) as time source, the drift is bigger, the stability of the clock rate is worse, and it is considered not to be suitable for applications that require granularity below one millisecond. Preliminary tests [21] show that the more suitable clock source for Timestamp generation is the HSE, as far as it provides a smaller period and more constant clock rate. However, it would also be possible to use the LSI as time source for monitoring environments where an accuracy of milliseconds could be acceptable, as discussed below.

For these experiments, the monitor nodes based on the STM32F4Discovery boards, were connected to an Ethernet network, which also includes a PC. This PC acted as both data collector and synchronization server (SyncRoot). The SyncRoot sent the synchronization signal periodically each *p* seconds. Two synchronization methods have been considered. The Sync signal may be sent via Ethernet, in a Synchronization frame, or through digital lines (used in preliminary tests), but results have proven that the choice has no influence on the precision of the proposal, whenever it complies with the requirements stated above. However, we decided to use Ethernet for these experiments, as this situation may be considered more realistic in a real distributed monitoring system. To avoid the delay that may be introduced by buffering operation in switched Ethernet networks, an Ethernet hub was used. When the SyncRoot sent a Sync frame, the hub transmitted it through all its ports without buffering or other delays. By using Ethernet and a hub, the end-to-end delay was kept constant and very small. It was verified that the SyncRoot only was sending the Sync frames, and no additional traffic.

An Ethernet-based implementation of this mechanism has been selected for these experiments because it is very useful, as far as the network is used for both synchronization messages throughout the monitoring campaign and trace collection after the end of the experiment. On the other hand, this approach is not always suitable for monitoring a deployed WSN, and it may only be possible in laboratory conditions.

The WSN to be observed was simulated by means of another STM32F4 Discovery board (on the left side of Figure 7). This device (called WSN Emulator) emulated the behavior of a set of WSN nodes running a temperature monitoring application. This WSN emulator generated the events that the monitor nodes had to register. For these experiments, each monitor node is connected to the WSN emulator by means of digital lines. One line per monitor was used in the first experiment (Figure 7), and two lines (one for each event: Transmission and Reception) were required in the second experiment.

### 5.2. First Experiment: Precision Test

#### 5.2.1. Scenario

Taking into account that the clock rate is not always constant in a node and can vary during time slots, as proven in previous tests, the first experiments were designed to evaluate the precision of the synchronization mechanism. In this scenario, the WSN Emulator was programmed to generate an event each 8 s and deliver it as a digital signal to six different monitor nodes at the same time. With this purpose, six digital lines of the same I/O port were wired to an interrupt line of the six monitor nodes. As far as all the lines are activated by the same operation of “write“ on the internal register of the microcontroller of this WSN emulator, and the length of cables is the same, no delay is expected between events detected by the monitor nodes.

Each monitor node created its own log, registering both the observed events and the synchronization signal with their timestamps. For the evaluation of the influence of the synchronization period in the precision of the corrected trace, many period times, from 5 s to 600 s were considered (5, 10, 30, 60, 120, 180, 240, 300, 450 and 600 s). Each test ran for about 60 min, and thus more than 500 events were registered.

Later, files containing these traces were sent to the Monitoring Server for Timestamp resynchronization using Equation (1) and analysis. In two series of experiments, both the external HSE crystal, provided by the expansion board STM32F4DIS-BB, and LSI RC oscillator, built into the microcontroller, were used as RTC source, and its results were compared. The limitations of the microcontroller provide a granularity of 40 μs per cycle when using the HSE, and 125 μs when using the LSI per cycle [35].

#### 5.2.2. Results

In this first set of experiments, the HSE clock source, with a low rate variability, was used as RTC. As defined above, the data of this experiment were processed and the traces obtained were synchronized using the Equation (1). In each experiment, approximately 500 events were detected and recorded in each one of all the six nodes. For each generated event, the six Timestamps obtained in the different monitor nodes were averaged, and the error was calculated as the deviation of each timestamp against this average. Table 1 shows the obtained results. The confidence interval of the error mean was obtained by Equation (2), where *S* is the standard deviation of the data obtained for each test, $\bar{x}$ is the mean of measurements (errors), *α* is the significance level (5%), and *n* is the number of measurements in the test:

(2) $$\bar{x}\pm t_{\alpha /2}\frac{s}{\sqrt{n}}$$

As it can be observed in Table 1, synchronization periods until 300 s guarantee an error below the RTC granularity. This means that these errors would not cause any difference in trace Timestamp. It cannot be assumed that, for periods above this value, the drift is constant along the period, and thus a non-negligible drift estimation error is suffered. This introduces an error in the Timestamp correction process, and may cause incorrect trace temporization.

As far as it is intended to grant the correct functioning of Timestamp resynchronization, period values greater than 300 s were not considered for next experiments, despite the fact that the Timestamp errors are very scarce, and its importance may be negligible as shown below.

**Figure 8:**
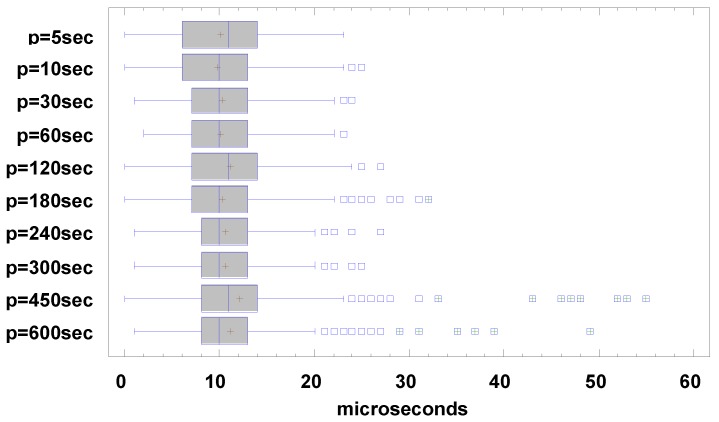
Boxplot graphic of the values obtained in the precision experiments.

Figure 8 shows the boxplot graphic of the distribution of the average error values obtained in each trace synchronization test for the different synchronization periods. The boxplot graphic illustrates the first and third quartiles—the gray rectangle—the mean—a red cross inside the rectangle—and the median—the blue vertical line in the rectangle—as the main statistical parameters. The outliers are represented with small squares outside the line, when these outliers affect the mean—e.g., for periods of 450 s and 600 s—a small cross inside the square is added. As expected, there is a tendency for higher error values to appear when using higher synchronization periods, some of them above the clock granularity. However, even with a long synchronization period (600 s), the obtained error results can be considered acceptable for application with weak or medium precision restrictions. The mean and the median are barely affected by the synchronization period. Nevertheless, as synchronization periods increase, higher error values are detected. Because of the reasons mentioned above, it is understandable—and even expected—that outliers appear, but these do not affect significantly the precision of the mechanism.

The average error of the proposed mechanism, when synchronization period values are not beyond 300 s, can be determined in 10 ± 1 µs. In order to compare the proposed mechanism with the so-called traditional synchronization method, the traces were been processed to simulate that behavior. They were modified, making the arrival of synchronization frame to cause all the nodes to set its internal clock with the same value than SyncRoot time. Then, the Timestamps of next events after synchronization were corrected accordingly. There has been assumed that no propagation delay existed, and all the nodes corrected their clocks exactly at the same time.

Table 2 shows the results of this test. As before, the values (mean error) are in microseconds and were obtained as the deviation of each Timestamp of the monitors for each recorded event. It can be seen that for a period of 5 s the values obtained are still acceptable, as the mean error is relatively low (16 µs) and errors are equal or lower than the RTC granularity, but they got worse with periods greater than 10 s. The mean and median for each synchronization period are very close. Therefore, this traditional synchronization method—internal clock synchronization—cannot provide itself the same precision for trace synchronization than our proposal—even with a highly precise clock source such as HSE—because the clock rate between the synchronization points is not corrected or adjusted.

**Figure 9:**
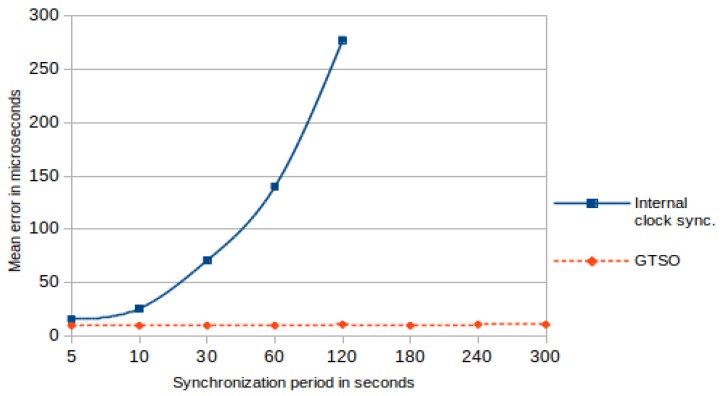
Mean error of internal clock synchronization and GTSO.

Figure 9 shows the graphical comparison of the mean error between the proposed trace synchronization mechanism and the traditional synchronization. This figure enhances the differences between the mean error values presented in Table 1 and Table 2. Whereas in the trace synchronization mechanism the mean error values are bounded between 10 and 12 μs or less, for traditional synchronization mechanism the mean error increases continuously, reaching to exceed 250 μs when the synchronization period was 120 s.

The second part of this experiment evaluated the incidence of the drift, clock rate variation and clock precision in the performance of the method. In this line, both of these mechanisms were reevaluated when using a low-quality clock source, such as the built-in LSI (32 KHz) as RTC source, with a high rate variability As previously described, the maximum precision grain that the microcontroller may obtain with this crystal is 125 μs, with a huge clock drift (it is based on a RC oscillator with no crystal). The conditions of these experiments were the same as the previously described experiments: the WSN emulator generated an event each 8 s, and the experiments ran for 60 min with synchronization periods varying from p = 5 s to p = 60 s. As discussed above, this clock source is not stable enough to provide precision more than at milliseconds level. That is why the error values below one millisecond, i.e., 1000 μs, have been considered as a success.

Table 3 shows the results of the precision tests after applying the GTSO mechanism. As stated above, the error was calculated as the deviation of each Timestamp with the mean of all the monitor nodes. When synchronization was frequent (period p = 5 s), the obtained values can be considered good, as error was below one millisecond in almost all measurements, with a mean error of about 0.4 ms. For greater synchronization periods, the clocks drift variations became more visible, and its effect over the precision must be considered relevant. When period p = 10 s, the values get worse slightly, but the mean error remains below the millisecond, as well as two-thirds of the values obtained. For longer synchronization periods, the results are not good, although they remain in the range of a few milliseconds. 

It can be seen that the results in Table 3 are worse than those shown in Table 1. This may be justified because of the low quality of LSI. Some of our previous experiments [21] had shown a drift up to 10% when the LSI was the timing source for the RTC. However, LSI was used in these tests for evaluating our proposal in the worst case. This may the only option when the hardware of the monitor nodes only offers this kind of RTC source.

Table 4 shows the results of the same test when traditional synchronization is applied. In all cases the mean error values obtained are about several milliseconds, even more than a second when period increases to p = 60s. In addition, the percentage of Timestamps that can be considered successful, that is, with an error below one millisecond, was less than the 1% of all the analyzed cases. Even more, the standard deviation is very high for all cases, indicating a high variability of the data obtained.

**Figure 10:**
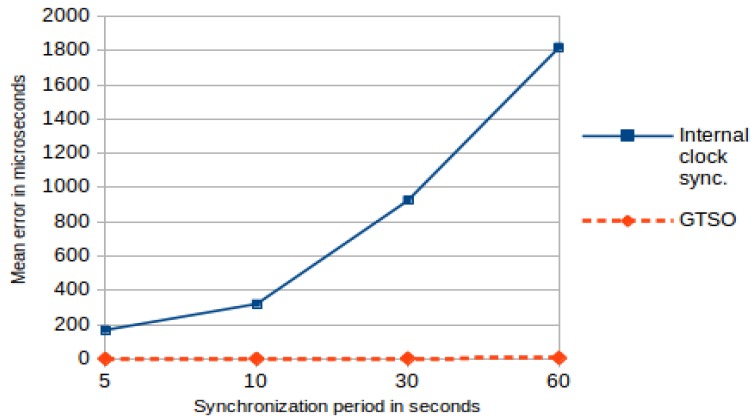
Mean error (arithmetic mean) of internal clock synchronization and GTSO, using the LSI as clock source.

Figure 10 shows a graphical comparison of the mean error of both tests using the LSI as the RTC source, for different periods of synchronization. These experiments enhance the fact that an internal clock synchronization approach is completely impossible when monitor nodes do not offer a high quality time source for the RTC, whereas our proposed trace synchronization mechanism would be used, and even obtain acceptable results, in these nodes if the synchronization period is low, around 5 s.

However, it is not recommended to use this type of clock source, especially for applications that require a thin grain or high precision.

### 5.3. Second Experiment: Sequence Tests

#### 5.3.1. Scenario

The second set of experiments evaluated the correctness of the sequence of events recorded in different monitor nodes, especially when an action sequence had been registered in different monitor nodes. For instance, let us consider the transmission of a frame between a source sensor node A and a destination sensor node B. Two related events should be generated in the monitoring platform: the monitor node of A should register a *Transmission event*, whereas the monitor node of B should register a *Received Frame event*. Both events are related to the same action—a frame transmission—and should be registered in order: Reception event in destination node should be registered a short time (corresponding with the transmission latency) later than the Transmission event in source node. 

**Figure 11:**
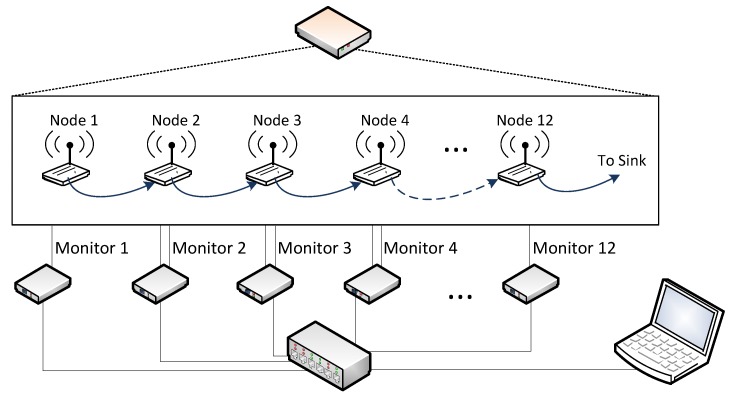
Scheme of the second experiment.

The hardware scheme of these experiments was similar that the presented in Figure 7: the WSN Emulator generated the events recorded by the monitor nodes. Moreover, each one of the previously described twelve virtual nodes is attached to its own monitor (Figure 11). The WSN Emulator ran a synthetic trace that simulated the behavior of the multi-hop routing algorithm of a WSN. This synthetic trace was based on the events obtained from a real capture campaign with real nodes. Figure 11 represents the simulated behavior of the WSN: Information was generated in node 1, and was sent to node 2 for routing, which also forwarded it to node 3. This multi-hop communication continued until the last node delivered the collected data to a sink.

In these experiments, a multi-hop communication from node 1 to the sink node is simulated. The transmitted data must be routed through nodes 2 to 12. This last node 12 finally delivers the data packet to the sink node. Each node *n* (from node 2 to node 11) generates two events, corresponding to the reception from the node *n − 1* and the transmission to the node *n + 1*. Node 1 will only generate a Transmission event, whereas node 12 only requires a Received Frame event. Thus, each monitor node—out of monitors 1 and 12—received two events to be recorded: *Reception* of the incoming frame and *Transmission* to the next hop in the routing path. Both events were recorded in the monitors in log files with its Timestamp for later synchronization. 

The performance of the monitoring platform has been evaluated for different values of the transmission period (p) of the synchronization signals. The used values have been from p = 30 s, 60 s, 120 s, 180 s, 240 s, and 300 s, that is, the values considered in the first series of experiment 1. Each scenario was simulated for 120 min, and more than 700 operating cycles of the entire WSN were observed. 

After the end of the experiment, all the trace files were delivered to the Monitor Server, and the aforementioned synchronization method was applied. The timestamps were recalculated, and for each pair of related events recorded in two different nodes—for example, Transmission event in sender node with Reception event in receiver one—the difference between Timestamps was calculated. This time should be positive—since the second event cannot occur before the first—and its value should be close to the simulated value in the synthetic trace.

#### 5.3.2. Results

The main objective of these experiments was the verification of the correctness of the order of the events after synchronization, especially when the same event had been registered in different nodes. It also allowed the quantification of the difference between Timestamps and real time data (time error) and its variability. These experiments also considered both the global trace synchronization proposal and the traditional synchronization mechanism described above. 

The synthetic trace used for the experiments includes a transmission time of 480 μs between transmission and reception events. This value was obtained by observation of a real WSN, with a transfer rate of 115.2 Kbps. With these times, the use of LSI was not considered acceptable, and thus HSE oscillator was used as RTC source. As previously mentioned, HSE offered a precision granularity of 40 μs. 

Therefore, the difference between the Timestamps of the transmission event (node *n − 1*) and the timestamp of reception event (at a node *n*) must be 480 ± 40 μs. This assumes that one cycle error in the RTC source in each pair of monitors, one positive and one negative, is introduced. 

Once the tests were performed, the obtained monitor’s data were processed. Table 5 summarizes the results obtained for GTSO. Although the median remains constant at the optimum value (480 μs), the mean error increases when the synchronization period is greater than *p* = 180 s, in a similar way to the standard deviation and the range of the confidence interval. The percentage of values within the range considered acceptable (480 ± 40μs) is above 93% up until a period *p* = 120 s, but begins to decrease from *p* = 180 s. Additionally, last column of Table 5 shows the percentage of erroneously ordered events, that is, the percentage of events that have a register a reception time less than the transmission time. Once again, due the clock rate is not always constant, there are values those affect the confidence interval and the percentage values in the accepted range of time difference.

From these results, it is possible to determine that, for optimal performance, the synchronization period be between 60 and 180 s, being the optimum 120 s. With these values, a mean error of 10 ± 1 μs is obtained. If the monitoring system allows a lower precision, a slightly longer period may be used, as shown before in Table 1.

Finally, Table 6 shows the results of the second test when applying the traditional internal clock synchronization mechanism. With this method only at p = 30 and 60 s the mean error and the median are kept at values close to 480 μs, but the percentage of values in the range 480 ± 40 μs does not reach 50%. From p = 120 s the values of the mean and median deviate from the optimal value, the percentage of values within the acceptable range decreases, and the confidence interval values increase. In all cases the standard deviation is relatively high, compared to global trace synchronization. Moreover, the percentage of erroneously ordered events demonstrates the inapplicability of traditional synchronization methods that only adjust the internal clocks.

**Figure 12:**
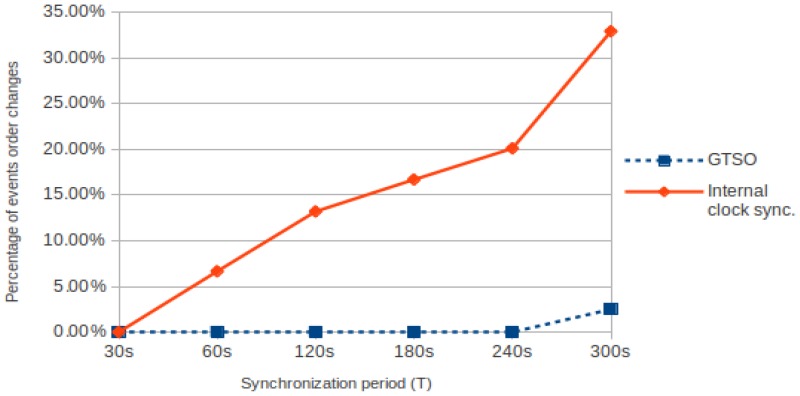
Events sequence errors (%) when applying both internal clock synchronization and GTSO.

Figure 12 shows the comparison of sequence errors (percentage), i.e., the changes in the order of the events, for both mechanisms. When internal clock is adjusted with traditional synchronization mechanisms, only for a period of *T* = 30 s the percentage is zero, but it increases until reaching almost 33% for *T* = 300 s. On the other hand—when global trace synchronization is used—the percentage is zero for almost all cases, with the exception of *T* = 300 s, where a 2.5% of the times the sequence registered is not correct. In this case, it is necessary to use a shorter synchronization period to avoid this kind of errors.

According to several proposals, the accuracy of a global traces synchronization method may be improved by decreasing the synchronization period, thus increasing the number of synchronization signals in the trace. This increases the workload to be assumed by the monitors (monitor and sniffer nodes, as well as SyncRoot). This approach has been used by other proposals—specially for online synchronization, as for example in [36,37,38]—which send the synchronization messages with higher frequency, even with a period of 1 s. In GTSO, which has been evaluated over a real monitoring platform under laboratory conditions, it can be seen that good precision and efficiency are achieved with a lower synchronization frequency, when offline synchronization is applied, despite the variation of drift. Besides, the synchronization period used can be directly related to the RTC clock source. On the other hand, GTSO does not depends of transmission-reception events to perform synchronizations, like others proposals such as [26,30,31]. This advantage let synchronize traces form monitoring nodes which do not transmit, e.g., sniffer nodes.

## 6. Conclusions 

In this work, a new mechanism for synchronizing WSN-DMP traces (GTSO) has been presented and evaluated. It consists of an offline post-facto mechanism based on the inclusion of global common events (synchronization points) in the traces of all the nodes (monitors and sniffers) of a monitoring platform. When the experimentation ends, a post-processing algorithm processes the traces by rearranging all events according to the synchronization points.

Results show that this simple mechanism achieves a correct behavior, for both precision and correct ordering. This way, the presented method guarantees an error lower than the clock granularity up to a synchronization period of 300 s when a crystal based RTC source is used. Almost 100% of the experiments have obtained an error less than 40 μs—the granularity of the RTC—with a mean error of about 10 μs. When using a cheaper RTC source, a RC oscillator (with a big lack of precision and frequency stability), which offers a very low performance, the proposal is still able to achieve a good behavior when using a high synchronization frequency. Obviously, external crystals with more precision and frequency stability permit better results, so a tradeoff between maximum granularity and a long synchronization period can be achieved for monitoring purposes when a good RTC source is used. This way, with synchronization periods of tens of seconds (and even hundreds), the overload caused by the presented mechanism is minimum. The SyncRoot just transmits the synchronization message that must be registered by all the nodes of the monitoring system. No other complex algorithm or computation is necessary while monitoring. 

Furthermore, the presented proposal offers many advantages in front of traditional (internal) clock synchronization methods, as they are usually focused to synchronize clocks or estimate the drift. Our proposal is oriented to maintain the correct sequence order of the registered events by a monitoring system of a DS. When dealing with causal-related events (e.g., an event in a node generates another event in other node), the sequence errors percentage was over the 50%. The proposed mechanism achieves a sequence error percentage below 10%. Additionally, traditional synchronization method requires a very small synchronization periods (below 5 s) to grant the correct sequence of events. In the same conditions, GTSO may ensure the correctness of the sequence of events for high synchronization periods, above 240 s. When considering also the computation time and number of transmitted messages, the convenience of using the global trace synchronization mechanism in front of the traditional synchronization schemes is very prominent.

Finally, the proposed trace synchronization mechanism GTSO may be applicable as far as a synchronization point may be issued to all the monitor and sniffer nodes with a bounded variability. Our previous experiments have proven a single digital line and Ethernet network for this end, but GTSO may suitable for different monitoring platforms which use other methods for including a synchronization point in the monitor node’s trace. The only restriction of GTSO is its dependence of a mechanism capable of sending the sync points adequately.

As future work, the authors are currently designing a wireless beacon system, which would be able to generate a common wireless synchronization signal in all the monitor nodes by transmitting it through a wireless band different from that used in the WSN. This would be made possible an easy deployment of our monitoring platform in applications with a huge number of sensor nodes, without further infrastructure requirements.

## Figures and Tables

**Table 1:** Results obtained in the precision experiment for GTSO (HSE as RTC source).

Period	Minimal Error (μs)	Maximal Error (μs)	Mean (μs)	Median (μs)	Standard Deviation	Confidence Interval at 95%	Percentage Less Than or Equal to 40 μs
**p = 5 s**	0	23	10	11	5.31	10.11 ± 0.44	100.0%
**p = 10 s**	0	25	10	10	4.97	9.80 ± 0.42	100.0%
**p = 30 s**	1	24	10	10	4.63	10.22 ± 0.39	100.0%
**p = 60 s**	1	23	10	10	4.34	10.12 ± 0.37	100.0%
**p = 120 s**	0	27	11	11	4.64	11.09 ± 0.40	100.0%
**p = 180 s**	0	31	10	10	4.71	10.29 ± 0.40	100.0%
**p = 240 s**	1	27	11	10	4.85	10.56 ± 0.41	100.0%
**p = 300 s**	1	25	11	10	5.23	10.54 ± 0.43	100.0%
**p = 450 s**	0	55	12	11	7.02	12.05 ± 0.61	98.3%
**p = 600 s**	1	49	11	10	5.59	11.18 ± 0.48	99.4%

**Table 2:** Results obtained in the precision experiment, for internal clock synchronization.

Period	Minimal Error (μs)	Maximal Error (μs)	Mean (μs)	Median (μs)	Standard Deviation	Percentage Less Than or Equal to 40 μs
**p = 5 s**	0	40	16	15	9	100.0%
**p = 10 s**	0	60	26	30	14	88.6%
**p = 30 s**	0	150	71	70	39	31.1%
**p = 60 s**	0	285	140	140	80	15.5%
**p = 120 s**	0	575	277	265	161	6.8%

**Table 3:** Results obtained in the precision experiment with the LSI as clock source, for the global traces synchronization.

Period	Minimal Error (μs)	Maximal Error (μs)	Mean (μs)	Median (μs)	Standard Deviation	Percentage Less Than or Equal to 1000 μs
**p = 5 s**	0	1204	397	363	238	98.40%
**p = 10 s**	41	2858	826	779	458	66.49%
**p = 30 s**	52	9353	2588	2460	1574	16.76%
**p = 60 s**	0	86,917	5519	5242	4544	5.72%

**Table 4:** Results obtained in the precision experiment using the LSI as clock source, simulating a clock synchronization process.

Period	Minimal Error (μs)	Maximal Error (μs)	Mean (μs)	Median (μs)	Standard Deviation	Percentage Less Than or Equal to 1000 μs
**p = 5 s**	0	303,190	168,688	169,490	235,771	0.71%
**p = 10 s**	6104	612,083	320,838	320,885	176,321	0.00%
**p = 30 s**	6990	1,867,750	925,267	935,208	543,465	0.00%
**p = 60 s**	0	3,685,792	1,819,828	1,820,896	1,068,465	0.36%

**Table 5:** Results obtained in the sequence experiment with GTSO.

Period	Mean	Median	Standard Deviation	Confidence Interval at 95%	Percentage in Range 440–520 μs	Change of Order (%)
**p = 30 s**	481	480	22.31	480.96 ± 1.01	93.48%	0.00%
**p = 60 s**	481	480	21.85	480.60 ± 0.97	93.65%	0.00%
**p = 120 s**	482	482	27.29	482.03 ± 1.21	93.35%	0.00%
**p = 180 s**	498	483	184.91	497 ± 11.21	91.48%	0.00%
**p = 240 s**	518	482	483.88	517.69 ± 22.06	90.23%	0.00%
**p = 300 s**	774	479	1848.42	774.40 ± 129.97	85.71%	2.50%

**Table 6:** Results obtained in the sequence experiment with tradition clock synchronization.

Period	Mean (μs)	Median (μs)	Standard Deviation	Confidence Interval at 95%	Percentage in Range 440–520 μs	Change of Order (%)
**p = 30 s**	485	480	122.16	485.39 ± 5.54	47.06%	0.00%
**p = 60 s**	490	480	269.01	490.09 ± 11.92	32.23%	6.65%
**p = 120 s**	501	520	559.70	500.784 ± 24.91	17.01%	13.20%
**p = 180 s**	710	520	746.77	710.21 ± 44.91	12.52%	16.69%
**p = 240 s**	719	520	1140.44	719.36 ± 117.73	12.70%	20.10%
**p = 300 s**	1446	440	11,250.0	1446.24 ± 682.09	11.96%	32.92%
